# Factors associated with the development of ocular candidiasis and ocular prognosis with echinocandin therapy for candidemia

**DOI:** 10.1186/s12348-021-00248-0

**Published:** 2021-06-14

**Authors:** Daiki Sakai, Wataru Matsumiya, Sentaro Kusuhara, Makoto Nakamura

**Affiliations:** 1 Department of Surgery, Division of Ophthalmology, Kobe University Graduate School of Medicine, 7-5-2 Kusunoki-cho, Chuo-ku, Kobe, 650-0017 Japan; 2 Department of Ophthalmology, Kobe City Eye Hospital, Kobe, Japan

**Keywords:** Ocular candidiasis, Endogenous endophthalmitis, Endophthalmitis, Chorioretinitis, Candidemia, *Candida albicans*, Echinocandins

## Abstract

**Purpose:**

To evaluate the factors associated with the development of ocular candidiasis (OC) and ocular prognosis with echinocandin therapy for candidemia.

**Methods:**

The medical records of 56 consecutive patients with a positive blood culture for *Candida* species between November 2016 and October 2019 were retrospectively reviewed. Information on patient characteristics, isolated *Candida* species, treatment details for candidemia, and ocular findings were extracted to identify factors associated with OC development.

**Results:**

The leading pathogen of candidemia was *Candida albicans (C.albicans)* (41.1%). Of 56 patients, 18 (32.1%) were diagnosed with chorioretinitis, categorized as either probable (8 patients) or possible OC (10 patients). There was no case of endophthalmitis with vitritis. The incidence of probable OC was not significantly different between the groups treated with echinocandins and other antifungal drugs (15.2% vs. 11.1%, *p* = 1.00). In all probable OC cases, systemic antifungal therapy was switched from echinocandins to azoles, and no case progressed to endophthalmitis. A multivariate logistic analysis revealed that female sex (adjusted odds ratio [aOR], 8.93; 95% confidence interval [CI], 1.09–72.9) and *C. albicans* (aOR, 23.6; 95% CI, 1.8–281) were independent factors associated with the development of probable OC.

**Conclusion:**

One-seventh of patients with candidemia developed probable OC. Given the evidence of female and *C. albicans* as the factors associated with OC development, careful ophthalmologic management is required with these factors, especially in candidemia. Although echinocandins had no correlation with OC development and did not lead to the deterioration of ocular prognosis, further investigation is required.

**Supplementary Information:**

The online version contains supplementary material available at 10.1186/s12348-021-00248-0.

## Key message

Previously some studies reported risk factors for the development of ocular candidiasis (OC), including hemodialysis, immunosuppressive status, and *C. albicans* involvement. In recent years, the ratio of *C. albicans* as the responsible species for candidemia has decreased and the use of echinocandins as initial therapy for candidemia has increased, which has a concern of effectiveness for patients with OC due to the poor concentration in vitreous fluid. In the present study with eight probable OC cases of 56 candidemia cases, we showed that the presence of *C. albicans* and being female were significant factors associated with OC development. In addition, initial therapy of echinocandins for candidemia had no correlation with OC development and did not lead to the deterioration of ocular prognosis.

## Introduction

Without early diagnosis and appropriate treatment, candidemia is a potentially fatal bloodstream infection worldwide [1]. The incidence of candidemia is growing alongside the increasing patient population at higher risk of infection due to the use of immunosuppressive drugs, aggressive chemotherapy, broad-spectrum antibiotics, and intravenous devices [2, 3]. Although *Candida albicans* (*C.albicans*) remains the most common species responsible for candidemia, a shift toward *Candida* non-*albicans* species has been reported in the recent years [2–4]. With regard to candidemia, there have been recent trends in increasing echinocandin use [5] and prompt initiation of antifungal therapy [6]. The Infectious Diseases Society of America (IDSA) currently recommends echinocandin drugs for the initial treatment of candidemia and invasive candidiasis [7]. Echinocandins have emerged as preferred agents for most episodes of candidemia and invasive candidiasis, except for the involvement of the central nervous system, eye, and urinary tract, where they can achieve poor concentration. This preference is based on a strong safety profile, convenience, early fungicidal activity, and a trend toward better outcomes based on data from individual studies and combined analyses of candidemia studies [8, 9].

Ocular candidiasis (OC) is an important disseminated complication of candidemia. The incidence of OC in patients with candidemia reported in previous studies is quite variable, ranging from 2.9% to 44.7% [10–19]. To date, several studies have reported risk factors for the development of OC, such as hemodialysis, parenteral hyperalimentation, immunosuppression, and *C. albicans* involvement [11, 13, 20]. One of the topics is the potential concern that initial systemic antifungal therapy with echinocandins might increase OC development because of poor intraocular penetration. Systemic echinocandins are known to have limited ability to penetrate the vitreous humor [21, 22]. Therefore, the IDSA does not recommend echinocandin use for *Candida* endophthalmitis [7]. Although some studies have reported that there is no evidence that initial echinocandins increase the incidence of OC [20, 23], whether initial echinocandins are an associated factor for the development of OC remains under debate. In addition, no report has clarified whether echinocandin use has a potential impact on ocular prognosis in candidemia.

The epidemiology and management of candidemia is constantly changing, alongside the evolution of medical technology; however, the impact of recent trends on the development and prognosis of OC has not been fully evaluated. This study aims to evaluate the clinical prognostic factors for the development of OC in patients with candidemia and the relationship between ocular prognosis and echinocandins therapy for candidemia.

## Methods

### Study design and setting

This retrospective study was performed according to the Declaration of Helsinki and was approved by the medical ethics committee at the Kobe University Hospital (Kobe, Japan). Because this was an observational study involving the use of medical records, the committee waived the requirement for informed consent. The confidentiality of patient data shall be maintained. We retrospectively reviewed the medical records of consecutive patients who were referred to the department of ophthalmology for positive blood culture of *Candida* species between November 2016 and October 2019. If there were multiple consultations for a single patient during the same hospitalization, only the first one was included. Patients whose medical records did not include the presence of ocular involvement or details of initial antifungal therapy were excluded.

We used the criteria described below to classify the ocular findings in accordance with previous studies [15, 17]. Proven endophthalmitis was defined as ocular lesions with positive cultures of vitreous humor. Probable endophthalmitis was defined as vitritis, including a typical fluffy ball extending into the vitreous cavity. Probable chorioretinitis was defined as deep, focal, white lesions that were restricted to the chorioretinal layers. Possible chorioretinitis was defined as other nonspecific chorioretinal lesions including retinal hemorrhage, cotton wool spots, or Roth spots. Then, both probable endophthalmitis and probable chorioretinitis were categorized as probable OC and possible chorioretinitis was categorized as possible OC. When both eyes in a patient were affected, the eye with more severe OC was applied to analyses in the current study. We reviewed the patient characteristics, isolated *Candida* species, details of candidemia or OC treatment, and ocular findings. The incidence of OC was calculated as the number of patients with ocular findings that met the diagnostic criteria of OC mentioned above.

### Study objective

This study primarily aims to evaluate the factors associated with the development of probable OC in patients with candidemia. The following potential factors were included in multivariate logistic regression model: diabetes mellitus, malignancy (solid tumor or hematological tumor), collagen-related disease, heart failure, kidney failure, elevated liver enzyme, hypertension, anemia, thrombocytepenia, neutropenia, corticosteroid use, immunosuppressive drug use, recent systemic surgery (within the past 1 month), intravenous hyperalimentation, intravascular devices, history of ophthalmic surgery, and β-D-glucan blood level. Information about the terms of variable factors was collected from the medical records. Following specific conditions were also evaluated based on the laboratory data. Referring to the blood test results of the closest available date to blood culture collection, we defined kidney failure as creatinine level of 1 mg/dL or higher, anemia as hemoglobin level of 11 g/dL or lower, neutropenia as neutrophil count of less than 1500 /μL, thrombocytepenia as platelet count of less than 100,000 /μL. Elevated liver enzyme was defined as abnormal elevation of liver enzymes at least one of asparate transaminase (AST), alanine transaminase (ALT), gamma glutamyl transferase (GGT) or alkaline phosphatase (ALP) with the following upper limits [24]: AST, 35 IU/L; ALT, 56 IU/L; GGT, 85 IU/L; ALP, 133 IU/L. Corticosteroid and immunosuppressive drugs were regarded as drug use when the patients were treated with them over a week of systemic administration regardless of dosage. After these data were collected, each factor was analyzed as a mono-variable. Eventually, factors associated with probable OC were analyzed using a multivariate logistic regression model.

The secondary objective of this study was to evaluate the relationship between initial antifungal therapy (echinocandins vs. other antifungal drugs or timing of treatment initiation) and probable OC. To investigate the effect of the difference of initial therapy for OC, patients who underwent antifungal therapy were divided into two groups based on the initial treatment regimen with echinocandins or other antifungal drugs and the timing of treatment initiation within 2 days or after 3 days from positive blood culture collection, respectively.

### Statistical analysis

All statistical analyses were performed using SPSS for Windows software package, version 25 (SPSS Inc., Chicago, IL, USA). Fisher’s exact test was used to compare categorical variables. The factors associated with the development of probable OC were analyzed by logistic regression analysis. All variables with a *P* value of less than 0.2 on univariate analyses were included in the multivariate analyses as potentially relevant factors. A *P* value of less than 0.05 was considered statistically significant. In the statistical analysis, a blood β-D-glucan level less than the limit of detection was considered to be 3 pg/mL.

## Results

### Patient profile and incidence of OC

There were 58 ophthalmology consultations for patients with positive blood culture of *Candida* species between November 2016 and October 2019. One patient had lagophthalmos, and her fundus was invisible as a result of the corneal condition. Another patient had persistent infection of *C. parapsilosis* for about 1½ years at the time of referral. These two patients were excluded from the evaluation. Overall, 56 patients with a blood culture positive for *Candida* species were included in the study.

Table 1 presents the patients’ profiles. There were 38 male patients and 18 female patients. The mean (SD) age at presentation was 67.6 (17.1) years, with a range of 13–90 years. The mean (SD) and the median time from positive blood culture to referral were 5.5 (3.7) and 5 days, respectively, with a range of 0–24 days. Moreover, 44 of 56 (78.6%) patients referred to ophthalmology within 1 week from positive blood culture. Thirty-eight patients (67.9%) had a single fundus examination. Ocular findings meeting the criteria of any kind of OC were detected in 18 of 56 patients (32.1%). Among them, eight patients with probable chorioretinitis (14.3%) were categorized as probable OCs, and ten patients with possible chorioretinitis (17.9%) were categorized as possible OCs. There was no case of probable endophthalmitis. Because none of our patients underwent vitrectomy or vitreous sampling, there was also no proven endophthalmitis.

**Table 1:** Clinical characteristics of the patients with and without probable OC

	Overall	Patients with probable OC	Patients without probable OC	*P* value
	(*n* = 56)	(*n* = 8)	(*n* = 48)	
Age (years), mean (SD)	67.6 ± 17.1	65.9 (20.0)	69.3 (14.4)	0.759
Female, n (%)	18 (32.1)	6 (75.0)	12 (25.0)	0.013^†^
Malignancy, n (%)	25 (44.6)	5 (62.5)	20 (41.7)	0.282
Solid tumor	20 (35.7)	4 (50.0)	16 (33.3)	0.368
Hematological tumor	5 (8.9)	1 (12.5)	4 (8.3)	0.704
Diabetes mellitus, n (%)	18 (32.1)	3 (37.5)	15 (31.3)	0.727
Corticosteroids use, n (%)	14 (25.0)	3 (37.5)	11 (22.9)	0.384
Immunosuppressive drug use, n (%)	15 (26.8)	4 (50.0)	11 (22.9)	0.123^b^
Neutropenia, n (%)	5 (8.9)	1 (12.5)	4 (8.3)	0.704
Recent systemic surgery (within 1 month), n (%)	36 (64.3)	5 (62.5)	31 (64.6)	0.909
IVH	42 (75.0)	6 (75.0)	36 (75.0)	1.000
Intravascular devices	52 (92.9)	7 (87.5)	45 (93.8)	0.534
Blood β-D-glucan (pg/mL), mean (SD) (n = 36)	98.4 (161.5)			
Blood β-D-glucan > 20 pg/mL, n (%) (*n* = 36)	16 (44.4)	3 (60.0)	13 (41.9)	0.655
Heart failure, n (%)	12 (21.4)	1 (12.5)	11 (22.9)	0.514
Kidney failure, n (%)	21 (37.5)	2 (25.0)	19 (39.6)	0.436
Elevated liver enzymes, n (%)	38 (67.9)	4 (50.0)	34 (70.8)	0.252
Hypertension, n (%)	21 (37.5)	3 (37.5)	18 (37.5)	1.000
Anemia, n (%)	49 (87.5)	7 (87.5)	42 (87.5)	1.000
Thrombocytopenia, n (%)	24 (42.9)	2 (25.0)	22 (45.8)	0.282
Collagen-related disease, n (%)	7 (12.5)	1 (12.5)	6 (12.5)	1.000
Previous history of ophthalmic surgery, n (%)	16 (28.6)	1 (12.5)	15 (31.3)	0.299
*Candida* species, n (%)				
*Candida albicans*	23 (41.1)	7 (87.5)	16 (33.3)	0.018^a†^
*Candida parapsilosis*	14 (25.0)	1 (12.5)	13 (27.1)	0.392 ^a^
*Candida glabrata*	7 (12.5)	0 (0.0)	7 (14.6)	
*Candida tropicalis*	7 (12.5)	0 (0.0)	7 (14.6)	
*Candida krusei*	3 (5.4)	0 (0.0)	3 (6.3)	
Other *Candida* species	2 (3.6)	0 (0.0)	2 (4.2)	

*OC* Ocular candidiasis, *SD* Standard deviation, *IVH* Intravenous hyperalimentation

^a^ Compared with other *Candida* species

^b^ Identified as potentially relevant

† Statistically significant at the *P* < 0.05 level

### Isolated Candida species

The leading pathogens were *C. albicans* (23 cases, 41.1%), followed by *C. parapsilosis* (14 cases, 25.0%), *C. glabrata* and *C. tropicalis* (7 cases, 12.5% in each), *C. krusei* (3 cases, 5.4%), and other *Candida* species (2 cases, 3.6%).

### Characteristic features of patients with and without probable OC

Table 1 shows the characteristics of patients with and without probable OC. In 18 female patients, 6 females were among the 8 patients with probable OC (75.0%), which is a significantly higher proportion as compared with the 12 females among 48 patients without probable OC (25.0%; *P* = 0.013). Among the 15 patients who received an immunosuppressive drug prior to candidiasis, 4 were among the 8 patients with probable OC (50.0%), which was not statistically different as compared with 11 patients among 48 patients without probable OC (22.9%; *P* = 0.123). In 18 patients with diabetes mellitus, the rate of 3 of 8 patients with probable OC (37.5%) was not statistically different as compared with 15 of 48 patients without probable OC (31.3%; *P* = 0.727). In 23 patients with *C. albicans* infection, 7 cases in 8 patients with probable OC (87.5%) was significant higher rate as compared with 16 patients in 48 patients without probable OC (33.3%; *P* = 0.018).

### Factors associated with probable OC

Table 2 presents the results of the logistic regression analysis for potential factors associated with probable OC. Univariate analyses revealed that being female and *C. albicans* infection were significantly associated with an increased risk of probable PC (univariable odds ratio [OR], 9.0; 95% confidence interval [CI], 1.6–50.7; univariable OR, 14.0; 95% CI, 1.6–123.8, respectively), whereas the use of an immunosuppressive drug before candidiasis had a univariable OR of 3.364 with no significance (95% CI, 0.7–15.7). These factors were identified as potentially relevant by univariate analyses. Eventually, multivariate analyses revealed that being female (*P* = 0.041; adjusted OR, 8.93; 95% CI, 1.09–72.9) and *C. albicans* infection (*P* = 0.012; adjusted OR, 23.6; 95% CI, 1.98–281) were independent factors associated with probable OC.

**Table 2:** Results of logistic regression analysis of factors associated with the development of probable OC

Variable	Univariate analysis			Multivariate analysis		
	OR	95% CI	*P* value	aOR	95% CI	*P* value
Age per year	0.993	0.953–1.036	0.759			
Sex (female/male)	9.000	1.598–50.691	0.013^†^	8.927	1.093–72.944	0.041^†^
Malignancy	2.333	0.499–10.907	0.282			
Solid tumor	2.000	0.442–9.056	0.368			
Hematological tumor	1.571	0.153–16.182	0.704			
Diabetes mellitus	1.32	0.278–6.257	0.727			
Corticosteroids use	2.018	0.415–9.815	0.348			
Immunosuppressive drug use	3.364	0.721–15.701	0.123^a^	2.885	0.316–26.350	0.348
Neutropenia	1.571	0.153–16.182	0.704			
Recent systemic surgery (within 1 month)	0.914	0.194–4.301	0.909			
IVH	1.000	0.178–5.632	1.000			
Intravascular devices	0.467	0.042–5.140	0.534			
Blood β-D-glucan > 20 pg/mL	0.655	0.138–3.472	0.457			
Heart failure	0.481	0.053–4.34	0.514			
Kidney failure	0.509	0.093–2.79	0.436			
Elevated liver enzymes	0.412	0.09–1.881	0.252			
Hypertension	1.000	0.213–4.693	1.000			
Anemia	1.000	0.104–9.614	1.000			
Thrombocytopenia	0.394	0.072–2.152	0.282			
Collagen-related disease	1.000	0.104–9.614	1.000			
Previous history of ophthalmic surgery	0.314	0.035–2.787	0.299			
*Candida* species (*Candida albicans*/non-*albicans*)	14.000	1.583–123.79	0.018^†^	23.624	1.984–281.289	0.012^†^

*OC* Ocular candidiasis, *OR* Odds ratio, *aOR* adjusted OR, *CI* Confidence interval, *IVH* Intravenous hyperalimentation

^a^ Identified as potentially relevant

† Statistically significant at the *P* < 0.05 level

### Initial antifungal therapy

Of the 56 patients, 55 were treated with systemic antifungal at referral. The only patient who did not receive an antifungal was already treated with vancomycin for catheter-related blood stream infection upon *C. albicans* detection, and his general status was improving at that time. Overall, 46 patients (83.6%) were treated with echinocandins (micafungin was used in all cases); 8 patients (14.5%) were treated with fluconazole, and 1 patient was treated with liposomal amphotericin B (L-AMB) (1.8%). Micafungin was the most common choice of initial therapy, which was empirically selected in the patients with fungal infection suspicion or detection. Otherwise, fluconazole was selected based on results of pathogen identification and/or antibiotics susceptibility test in 6 patients. The bases of antifungal selection based on medical records were unclear in remaining 2 patients treated with fluconazole and 1 patient treated with L-AMB. The incidence of probable OC was not significantly different between the groups treated with echinocandins and others (7 of 46 [15.2%] vs. 1 of 9 [11.1%]; *P* = 1.00; Table 3).

**Table 3:** Relationship between initial antifungal therapy and the development of probable OC

	Patients with probable OC	Patients without probable OC
Antifungal (*n* = 55)		
Echinocandins (*n* = 46)	7 (15.2%)	39 (84.8%)
Others (*n* = 9)	1 (11.1%)	8 (88.9%)
Initiation from blood culture collection (*n* = 41)		
Within 2 days (*n* = 31)	6 (19.4%)	25 (80.6%)
After 3 days (*n* = 10)	2 (20.0%)	8 (80.0%)

*OC* Ocular candidiasis

There were 14 patients who underwent empiric therapy with systemic antifungal administration before detection of fungus in blood culture. The remaining 41 patients were initiated on antifungal therapy based on the detection of positive blood culture. Among these patients, the median time from blood culture collection to initiation of antifungal drugs was 2 days, with a range of 1–4 days, and 39 patients started treatment within the day of detection. There were 31 patients (75.6%) who started treatment within 2 days and 10 patients (24.4%) who started treatment after 3 days from positive blood culture collection. The incidence of probable OC was not significantly different between the groups that started treatment within 2 days and those who started after 3 days from positive blood culture collection (6 of 31 [19.4%] vs. 2 of 10 [20.0%]; *P* = 1.00; Table 3).

### Clinical course

We confirmed reversing ocular findings (four complete resolution and four incomplete resolution) in all cases of probable OC with systemic antifungal therapy. In seven of eight cases of probable OC who received micafungin as initial therapy, the antifungal drug was changed to fluconazole before or after the OC diagnosis. The bases of these antifungal switching were diagnosis of OC in 4 patients, results of pathogen identification and/or antibiotics susceptibility test (de-escalation) in 2 patients, and unknown in remaining 1 patient. Among the cases with possible OC, three showed improving ocular findings (two with complete resolution and one with incomplete resolution), three cases showed unchanged findings, and the remaining four cases did not undergo a follow-up examination. No cases of OC progressed to endophthalmitis with vitritis. Moreover, none of the patients required vitrectomy. The mean (SD) follow-up time was 90 (134) days for patients with probable OC and 11 (14) days for patients with possible OC. Data on visual acuity were available for two cases of probable OC, which showed improvement in vision. In other cases, the visual acuity was not correctly evaluated because of the patients’ critical status.

## Discussion

In this study, 8 of 56 patients had typical ocular findings that met the criteria of probable OC and 10 of 56 patients had nonspecific ocular findings of possible OC. We calculated the incidence of probable OC as 14.3%. Recently, using the same criteria, Ueda et al. [19] and Son et al. [20] reported that the incidence of OC, except for possible cases, was 12.8% and 16.7%, respectively, which was similar to the incidence rate in this study.

Recently, some studies [2, 4] have shown that a change in the distribution of *Candida* species from *C. albicans* to *Candida* non-*albicans* is responsible for candidemia. In a previous similar study, *C. albicans* was reported to be a leading pathogen of candidemia. Nagao et al. [16] reviewed the records of patients between 2005 and 2011, and detected *C. albicans* in 52.5% of patients. Son et al. [20] reviewed the records of patients between 2014 and 2017, and detected *C. albicans* in 42.2% of patients. In our study, the leading pathogen was *C. albicans*, which was detected in 41.1% of patients; this was similar to the recent report. As for the antifungal selection of initial treatment, a recent trend is an increase in echinocandins. Muñoz et al. [23] reviewed the records of patients between 2010 and 2011, and found that echinocandins were selected for 33.3% of patients. Meanwhile, Kato et al. [18] reviewed the records of patients between 2011 and 2016, and determined that echinocandins were selected for 80.1% of patients. In our study, echinocandins was selected for 83.6% of patients, the highest proportion among previous studies. Thus, our results show the latest clinical features of OC, which are reflected by an increase in echinocandin use as initial treatment for candidemia.

Multivariate logistic regression analysis revealed that *C. albicans* infection and female sex were independent factors associated with probable OC. Among 8 cases of probable OC, 7 had *C. albicans* involvement, which was considered to be a significant factor associated with OC, consistent with previous reports [16, 25]. Abe et al. [26] also reported that *C. albicans* might have greater risk for OC over other *Candida* species. In addition, Abe et al. reported that ocular inflammation was more severe because of the greater cytokine/chemokine release and concomitant recruitment of neutrophils and monocytes in mice with *C. albicans* infection as compared with *Candida* non-*albicans* infection. Some studies in the 2000s reported male sex as a predisposing factor for OC development. Although male was reported as the majority of the patients with OC in these studies [27, 28], the rationale for male as a predisposing factor was not clarified. More recent studies have reported that sex differences have no relevance [18, 20]. The current study is the first to show that female sex is a factor associated with probable OC development. The infection caused by *Candida* spp. affects 70%–75% of women at least once during their lives, and 10% of women have asymptomatic vaginal colonization with *Candida* spp. [29]. The presence of *Candida* in the vagina, in the absence of immunosuppression or damaged mucosa, is usually not associated with any signs of disease. However, one report showed confirmed *C. albicans* endogenous endophthalmitis in a 35-year-old diabetic female patient with a 1-year history of severe chronic vaginal *C. albicans* infection [30]. That case highlighted that a number of systemic predisposing factors, such as long-standing type 2 diabetes mellitus, a recent history of admission for urinary sepsis, a recent use of broad-spectrum systemic antibiotics, and topical dexamethasone therapy in the right eye, could result in endogenous endophthalmitis due to vaginal candidiasis. Vaginal candidiasis most frequently affects young women of childbearing age, whereas candiduria typically occurs in elderly, hospitalized, or immunocompromised patients [31]. Unless there are coexisting predisposing factors, candiduria does not generally lead to invasive candidiasis. However, Suzuki et al. reported a case of *C. albicans* endogenous endophthalmitis in a 51-year-old female patient with candiduria secondary to urinary tract infection (UTI) who had had poor control of her diabetes mellitus [32]. They suggested that performing frequent ophthalmologic examinations in patients with diabetes who have candiduria and symptoms of a UTI is crucial due to the possibility of developing candidemia. In the present study, as it is shown in Supplemental Table 1, among 7 female patients who had urine culture results, 5 patients (71.4%) had candiduria; and among 6 female patients whose results of urinalysis prior to antifungal treatment were available, 5 patients (83.3%) had leukocyteuria. The incidence of candiduria and leukocyteuria were higher in female than in male. Although the available data was limited, that might support that candiduria or UTI were relatively common conditions in female patients with candidemia. Moreover, 8 of 18 (44.4%) women had a history of steroid use, 9 of 18 (50.0%) women had a history of immunosuppressive drug use, and 7 of 18 (38.9%) women had diabetes mellitus (Supplemental Table 1). Thus, the female sex might be considered a potential factor associated with OC in the presence of certain conditions.

Based on their excellent safety profile and broad spectrum of activity [33], echinocandins are widely used as first-line therapy for candidemia. However, they are not recommended for patients with OC because of a concern regarding their poor intraocular penetration [7]. Given this pharmacological aspect of echinocandins, the utility of echinocandin therapy for ocular complications in candidemia is conflicting. Micafungin which is the representative drug of echinocandin, has poor penetration into the vitreous after a single intravenous injection in animal models, although concentrations in retina-choroid and plasma exceeded the minimal antifungal inhibitory concentrations for endophthalmitis [34]. Mochizuki et al., reported micafungin levels in blood, cornea, retina-choroid, aqueous humor, and vitreous humor in eight eyes of 7 patients with the fungal disease who received intravenous injections of 150 to 300 mg micafungin. Though the micafungin levels in all samples exceeded the MICs, the levels in the vitreous and aqueous humor were lower [35]. In addition, the mean vitreous levels of micafungin with endogenous endophthalmitis still remained low, suggesting that even with a disrupted blood-retinal barrier, micafungin penetrated poorly into the vitreous [36]. On the other hands, recently, some clinical studies have reported that there is no evidence of a risk for development of OC with echinocandin use for the initial treatment of candidemia [20, 23]. The current study also showed that there was no difference in the incidence of probable OC between the groups treated with echinocandins and others. In this study, 46 (83.6%) out of 55 patients received echinocandins as initial antifungal therapy, and then no patient developed endophthalmitis with vitritis. While, according to Son et al. [20] and Muñoz et al. [23] reports, 90 out of 275 patients (32.7%) and 56 out of 168 patients (33.3%) received echinocandins as initial antifungal therapy, then 8 (3.0%) and 2 (1.2%) patients developed endophthalmitis, respectively. Although there was a discrepancy in the proportion of initial use of echinocandins, the very low incidence of endophthalmitis in this study is consistent with results from prior studies [20, 23]. This might support the hypothesis that echinocandins as initial therapy were not associated with the development of endophthalmitis. In addition, no cases of probable OC progressed to endophthalmitis with vitritis, despite 7 of 8 cases receiving echinocandins as initial therapy. However, because of the limitations of available data, whether echinocandins were effective for *Candida* chorioretinitis without vitreal involvement was unclear. Although echinocandins were switched to fluconazole at time of diagnosis of OC or the de-escalation of antifungal therapy in all cases, our results suggest that, under appropriate ophthalmic evaluation, the initial use of echinocandins might not have a negative impact on the ocular prognosis in candidemia.

With regard to the timing of treatment initiation for candidemia, recent evidence supports the importance of earlier intervention with antifungal therapy [8, 37, 38]. Considering that the incubation time for candidemia was reported to be about 2 days [39], the median time of 2 days from blood culture collection to antifungal therapy initiation wouldmeanthat most patients received an early intervention for candidemia in our study. We found no significant difference in the incidence between the groups treated within 2 days and those treated after 3 days from positive blood culture collection, which was attributed to the relatively unified early intervention.

The major limitation of this study was the absence of predetermined criteria for ophthalmologic screening for patients with candidemia. Although most of the patients (78.6%) referred to ophthalmology within 1 week from positive blood culture, there might be potential cases with the ocular findings which had already disappeared at the timing of ophthalmological examination in this study. Moreover, the majority of the patients (67.9%) had only a single fundus examination, and some of them could develop OC after an examination. Furthermore, we included only cases with candidemia who were referred to the department of ophthalmology. The rate of ophthalmological assessment among patients with candidemia has been reported to range from 46% to 71.8% [19, 21, 40]. Thus, we might underestimate the incidence of OC. Other limitations included the retrospective nature of the study design and the relatively small sample size. Our study had eight cases of probable OC, which may have limited the statistical power. Finally, the evaluation of visual acuity was limited because of the critical status of most of the patients in this study. We expected future studies involving large sample sizes with unified early ophthalmology referral and multiple ophthalmological examinations to validate our findings in this study. Additionally, in the current study, echinocandins were changed to fluconazole in all cases of probable OC before or after diagnosis. This fact might have made a considerable contribution to the preferable prognosis. Further studies that include an increased number of cases which treated with echinocandins are needed to investigate the impact of echinocandin use for OC management, including its incidence and prognosis. The investigation in this study was mainly performed for probable OC, because possible OC might include ocular lesions, which have different etiologies from *Candida* infection, such as ischemia and hypertension, leading to a concern of overdiagnosis among patients with possible OC [19].

In conclusion, probable OC was developed in about one-seventh of patients with candidemia. The presence of *C. albicans* infection and female sex could be significant factors associated with the development of OC in patients with candidemia. Therefore, careful ophthalmologic management might be recommended for patients with candidemia, especially in female patients or those patients with *C. albicans* involvement. Echinocandins had no correlation with OC development. In addition, when an alternative therapy of echinocandins is appropriately considered at the time of diagnosis of OC or with de-escalation of antifungal therapy, the initial use of echinocandins might not be associated with any concerns regarding ocular prognosis.

## Supplementary Information

**Additional file 1: Supplemental Table 1.** Clinical characteristics of female and male patients.

## Data Availability

All data included in this study are available from the corresponding author upon reasonable request.

## References

[1] Hassan I, Powell G, Sidhu M, Hart WM, Denning DW (2009). Excess mortality, length of stay and cost attributable to candidaemia. J Inf Secur.

[2] Papadimitriou-Olivgeris M, Spiliopoulou A, Kolonitsiou F, Bartzavali C, Lambropoulou A, Xaplanteri P, Anastassiou ED, Marangos M, Spiliopoulou I, Christofidou M (2019). Increasing incidence of candidaemia and shifting epidemiology in favor of Candida non-albicans in a 9-year period (2009–2017) in a university Greek hospital. Infection.

[3] Raja NS (2020). Epidemiology, risk factors, treatment and outcome of Candida bloodstream infections because of Candida albicans and Candida non-albicans in two district general hospitals in the United Kingdom. Int J Clin Pract.

[4] Goemaere B, Becker P, Wijngaerden EV (2018). Increasing candidaemia incidence from 2004 to 2015 with a shift in epidemiology in patients preexposed to antifungals. Mycoses.

[5] Mencarini J, Mantengoli E, Tofani L, Riccobono E, Fornaini R, Bartalesi F, Corti G, Farese A, Pecile P, Boni L, Rossolini GM, Bartoloni A (2018). Evaluation of candidemia and antifungal consumption in a large tertiary care Italian hospital over a 12-year period. Infection.

[6] Kollef M, Micek S, Hampton N, Doherty JA, Kumar A (2012). Septic shock attributed to Candida infection: importance of empiric therapy and source control. Clin Infect Dis.

[7] Pappas PG, Kauffman CA, Andes DR, Clancy CJ, Marr KA, Ostrosky-Zeichner L, Reboli AC, Schuster MG, Vazquez JA, Walsh TJ, Zaoutis TE, Sobel JD (2016). Clinical practice guideline for the management of candidiasis: 2016 update by the Infectious Diseases Society of America. Clin Infect Dis Off Publ Infect Dis Soc Am.

[8] Andes DR, Safdar N, Baddley JW, Playford G, Reboli AC, Rex JH, Sobel JD, Pappas PG, Kullberg BJ, for the Mycoses Study Group (2012). Impact of treatment strategy on outcomes in patients with candidemia and other forms of invasive candidiasis: a patient-level quantitative review of randomized trials. Clin Infect Dis.

[9] Mora-Duarte J, Betts R, Rotstein C, Colombo AL, Thompson-Moya L, Smietana J, Lupinacci R, Sable C, Kartsonis N, Perfect J (2002). Comparison of caspofungin and amphotericin B for invasive candidiasis. N Engl J Med.

[10] Henderson DK, Edwards JE, Montgomerie JZ (1981). Hematogenous Candida Endophthalmitis in patients receiving parenteral hyperalimentation fluids. J Infect Dis.

[11] Parke DW, Jones DB, Gentry LO (1982). Endogenous endophthalmitis among patients with Candidemia. Ophthalmology.

[12] Bross J, Talbot GH, Maislin G, Hurwitz S, Strom BL (1989). Risk factors for nosocomial candidemia: a case-control study in adults without leukemia. Am J Med.

[13] Donahue SP, Greven CM, Zuravleff JJ, Eller AW, Nguyen MH, Peacock JE, Wagener MM, Yu VL (1994). Intraocular candidiasis in patients with Candidemia: clinical implications derived from a prospective multicenter study. Ophthalmology.

[14] Shah CP, McKey J, Spirn MJ, Maguire J (2008). Ocular candidiasis: a review. Br J Ophthalmol.

[15] Oude Lashof AML, Rothova A, Sobel JD, Ruhnke M, Pappas PG, Viscoli C, Schlamm HT, Oborska IT, Rex JH, Kullberg BJ (2011). Ocular manifestations of Candidemia. Clin Infect Dis.

[16] Nagao M, Saito T, Doi S, Hotta G, Yamamoto M, Matsumura Y, Matsushima A, Ito Y, Takakura S, Ichiyama S (2012). Clinical characteristics and risk factors of ocular candidiasis. Diagn Microbiol Infect Dis.

[17] Tanaka H, Ishida K, Yamada W, Nishida T, Mochizuki K, Kawakami H (2016). Study of ocular candidiasis during nine-year period. J Infect Chemother Off J Jpn Soc Chemother.

[18] Kato H, Yoshimura Y, Suido Y, Ide K, Sugiyama Y, Matsuno K, Nakajima H (2018). Prevalence of, and risk factors for, hematogenous fungal endophthalmitis in patients with Candida bloodstream infection. Infection.

[19] Ueda T, Takesue Y, Tokimatsu I, Miyazaki T, Nakada-Motokawa N, Nagao M, Nakajima K, Mikamo H, Yamagishi Y, Kasahara K, Yoshihara S, Ukimura A, Yoshida K, Yoshinaga N, Izumi M, Kakeya H, Yamada K, Kawamura H, Endou K, Yamanaka K, Yoshioka M, Amino K, Ikeuchi H, Uchino M, Miyazaki Y (2019). The incidence of endophthalmitis or macular involvement and the necessity of a routine ophthalmic examination in patients with candidemia. PLoS One.

[20] Son H-J, Kim MJ, Lee S, Choi S, Jung KH, Jung J, Chong YP, Kim SH, Choi SH, Kim YS, Woo JH, Lee JY, Lee SO (2019). Risk factors and outcomes of patients with ocular involvement of candidemia. PLoS One.

[21] O’Day DM, Head WS, Robinson RD (1985). Intraocular penetration of systemically administered antifungal agents. Curr Eye Res.

[22] Gauthier GM, Nork TM, Prince R, Andes D (2005). Subtherapeutic ocular penetration of Caspofungin and associated treatment failure in Candida albicans Endophthalmitis. Clin Infect Dis.

[23] Muñoz P, Vena A, Padilla B, Valerio M, Sanchez MI, Puig-Asensio M, Fortún J, Fernández-Ruiz M, Merino P, Losa JE, Loza A, Rivas RA, Bouza E, CANDIPOP Project, GEIH-GEMICOMED (SEIMC), and REIPI (2017). No evidence of increased ocular involvement in candidemic patients initially treated with echinocandins. Diagn Microbiol Infect Dis.

[24] Gowda S, Desai PB, Hull VV (2009). A review on laboratory liver function tests. Pan Afr Med J.

[25] Lingappan A, Wykoff CC, Albini TA, Miller D, Pathengay A, Davis JL, Flynn HW (2012). Endogenous fungal endophthalmitis: causative organisms, management strategies, and visual acuity outcomes. Am J Ophthalmol.

[26] Abe M, Kinjo Y, Ueno K, Takatsuka S, Nakamura S, Ogura S, Kimura M, Araoka H, Sadamoto S, Shinozaki M, Shibuya K, Yoneyama A, Kaku M, Miyazaki Y (2018) Differences in ocular complications between Candida albicans and non-albicans Candida infection analyzed by epidemiology and a mouse Ocular Candidiasis Model. Front Microbiol 9. 10.3389/fmicb.2018.02477 10.3389/fmicb.2018.02477 PMC6199462 30386320

[27] Tanaka M, Kobayashi Y, Takebayashi H, Kiyokawa M, Qiu H (2001). Analysis of predisposing clinical and laboratory findings for the development of endogenous fungal endophthalmitis. A retrospective 12-year study of 79 eyes of 46 patients. Retina Phila Pa.

[28] Takebayashi H, Mizota A, Tanaka M (2006). Relation between stage of endogenous fungal endophthalmitis and prognosis. Graefes Arch Clin Exp Ophthalmol Albrecht Von Graefes Arch Klin Exp Ophthalmol.

[29] Sobel JD (2007). Vulvovaginal candidosis. Lancet.

[30] Hassan A, Poon W, Baker M, Linton C, Mühlschlegel FA (2012). Confirmed Candida albicans endogenous fungal endophthalmitis in a patient with chronic candidiasis. Med Mycol Case Rep.

[31] Achkar JM, Fries BC (2010). Candida infections of the genitourinary tract. Clin Microbiol Rev.

[32] Suzuki R, Kuroda H, Matsubayashi H, Ishii A, Toyoda F, Kawarai Lefor A, Sugawara H (2015). Candidemia from an upper urinary tract infection complicated by candida endophthalmitis. Intern Med Tokyo Jpn.

[33] Denning DW (2003). Echinocandin antifungal drugs. Lancet.

[34] Suzuki T, Uno T, Chen G, Ohashi Y (2008). Ocular distribution of intravenously administered micafungin in rabbits. J Infect Chemother Off J Jpn Soc Chemother.

[35] Mochizuki K, Sawada A, Suemori S, Kawakami H, Niwa Y, Kondo Y, Ohkusu K, Yamada N, Ogura S, Yaguchi T, Nishimura K, Kishino S (2013). Intraocular penetration of intravenous micafungin in inflamed human eyes. Antimicrob Agents Chemother.

[36] Moyer AL, Ramadan RT, Novosad BD, Astley R, Callegan MC (2009). Bacillus cereus-induced permeability of the blood-ocular barrier during experimental endophthalmitis. Invest Ophthalmol Vis Sci.

[37] Ostrosky-Zeichner L, Kullberg BJ, Bow EJ, Hadley S, León C, Nucci M, Patterson TF, Perfect JR (2011). Early treatment of candidemia in adults: a review. Med Mycol.

[38] Grim SA, Berger K, Teng C, Gupta S, Layden JE, Janda WM, Clark NM (2012). Timing of susceptibility-based antifungal drug administration in patients with Candida bloodstream infection: correlation with outcomes. J Antimicrob Chemother.

[39] Nunes CZ, Marra AR, Edmond MB, da Silva VE, Pereira CAP (2013). Time to blood culture positivity as a predictor of clinical outcome in patients with Candida albicansbloodstream infection. BMC Infect Dis.

[40] Takesue Y, Ueda T, Mikamo H, Oda S, Takakura S, Kitagawa Y, Kohno S, Masuda A, Yoshida C, Yasunaga C, Yamashita C, Nakataki E, Ohyagi H, Yagi H, Johnai H, Murai H, Hanamoto H, Nakamura I, Sanada I, Tandai I, Kuroki J, Ogawa J, Kawahara K, Amino K, Nakajima K, Yoshimoto K, Takeda K, Nakamura K, Suzuki K, Yamada K, Aizawa M, Hashimoto M, Ogata M, Shirano M, Kawada M, Kaneda M, Yoshioka M, Okuda N, Sugita N, Kikuchi N, Fuke S, Tsuchihashi S, Sugitani S, Ikuta S, Honda S, Nei T, Iwamura T, Yagi T, Kaji T, Ichimiya Y, Kobayashi Y, Minamishima Y, Goto Y, Hatano Y, Nagao Y, Yamagishi Y, Sashihara J, Tsukamoto A, Kawaoka T, Kobayashi M, on behalf of the ACTIONs Project (2015). Management bundles for candidaemia: the impact of compliance on clinical outcomes. J Antimicrob Chemother.

