# Supplementary material for: Factors associated with the development of ocular candidiasis and ocular prognosis with echinocandin therapy for candidemia
Source: J Ophthalmic Inflamm Infect. 2021 Jun 14;11:17. doi: 10.1186/s12348-021-00248-0 (PMC8200333; doi:10.1186/s12348-021-00248-0)
Supplement: Supplementary file 1 — Additional file 1: Supplemental Table 1. Clinical characteristics of female and male patients. [file 12348_2021_248_MOESM1_ESM.docx]

**Title:**

Factors associated with the development of ocular candidiasis and ocular prognosis with echinocandin therapy for candidemia

**Journal:** Journal of Ophthalmic Inflammation and Infection

Daiki Sakai, MD^1,2^; Wataru Matsumiya, MD, PhD^1^; Sentaro Kusuhara, MD, PhD^1^; Makoto Nakamura, MD, PhD^1^

^1)^Department of Surgery, Division of Ophthalmology, Kobe University Graduate School of Medicine, Kobe, Japan

^2)^Department of Ophthalmology, Kobe City Eye Hospital, Kobe, Japan

**Corresponding author:**

Wataru Matsumiya, MD, PhD

Department of Surgery, Division of Ophthalmology,

Kobe University Graduate School of Medicine.

7-5-2 Kusunoki-cho, Chuo-ku, Kobe 650-0017, Japan.

Tel +81-78-382-6048, Fax +81-78-382-6059

E-mail: [ytkmatsu@hotmail.com](mailto:ytkmatsu@hotmail.com)

**Supplemental Table 1** Clinical characteristics of female and male patients

|  | Female (n = 18) | Male (n = 38) |
| --- | --- | --- |
| Age (years), mean (SD) | 68.9 (18.3) | 67.0 (16.9) |
| Malignancy, n (%) | 9 (50.0) | 16 (42.1) |
| Solid tumor | 6 (33.3) | 14 (36.8) |
| Hematological tumor | 3 (16.7) | 2 (5.3) |
| Diabetes mellitus, n (%) | 7 (38.9) | 11 (28.9) |
| Corticosteroids use, n (%) | 8 (44.4) | 6 (15.8) |
| Immunosuppressive drug use, n (%) | 9 (50.0) | 6 (15.8) |
| Neutropenia, n (%) | 3 (16.7) | 2 (5.3) |
| Recent systemic surgery (within 1 month), n (%) | 8 (44.4) | 28 (73.7) |
| IVH | 14 (77.8) | 28 (73.7) |
| Intravascular devices | 17 (94.4) | 35 (92.1) |
| Blood β-D-glucan >20 pg/mL, n (%) (n = 36) | 4 (50.0) | 12 (42.9) |
| Heart failure, n (%) | 3 (16.7) | 9 (23.7) |
| Kidney failure, n (%) | 5 (27.8) | 16 (42.1) |
| Elevated liver enzymes, n (%) | 12 (66.7) | 26 (68.4) |
| Hypertension, n (%) | 7 (38.9) | 14 (36.8) |
| Anemia, n (%) | 15 (83.3) | 34 (89.5) |
| Thrombopenia, n (%) | 8 (44.4) | 16 (42.1) |
| Collagen disease, n (%) | 3 (16.7) | 4 (10.5) |
| Previous history of ophthalmic surgery, n (%) | 5 (27.8) | 11 (28.9) |
| *Candida* species, n (%) |  |  |
| *C. albicans* | 8 (44.4) | 15 (39.5) |
| *C. parapsilosis* | 5 (27.8) | 9 (23.7) |
| *C. glabrata* | 2 (11.1) | 5 (13.2) |
| *C. tropicalis* | 1 (5.6) | 6 (15.8) |
| *C. krusei* | 2 (11.1) | 1 (2.6) |
| Other *Candida* species | 0 (0.0) | 2 (5.3) |
| Candiduria, n (%) | 5 (71.4)^*^ | 5 (50.0)^**^ |
| Leukocyteuria, n (%) | 5 (83.3)^†^ | 1. (66.7)^††^ |

*SD* standard deviation, *IVH* intravenous hyperalimentation * Female (n=7), **Male (n=10), † Female (n=6), †† Male (n=6).
